# Transfer of marine mercury to mountain lakes

**DOI:** 10.1038/s41598-017-13001-2

**Published:** 2017-10-05

**Authors:** Sophia V. Hansson, Jeroen Sonke, Didier Galop, Gilles Bareille, Séverine Jean, Gaël Le Roux

**Affiliations:** 1 EcoLab, Université de Toulouse, CNRS, INPT, UPS, Avenue de l’Agrobiopole, 31326 Castanet Tolosan, France; 2 Observatoire Midi-Pyrénées, Laboratoire Géosciences Environnement Toulouse, CNRS; IRD, Université de Toulouse, 14, Avenue Édouard Belin, 31400 Toulouse, France; 3 Laboratory GEODE UMR 5602, Labex DRIIHM (OHM Haut Vicdessos), CNRS, Université J. Jaurès, 5 Allée A. Machado, 31058 Toulouse, France; 4 CNRS/Univ Pau & Pays Adour, Institut des Sciences Analytiques et de Physico-Chimie pour l’Environnement et les Matériaux – UMR5254, 64000 Pau, France; 5 Present Address: Department of Bioscience, Aarhus University, Fredriksborgvej 399, 4000 Roskilde, Denmark

## Abstract

Stocking is a worldwide activity on geographical and historical scales. The rate of non-native fish introductions have more than doubled over the last decades yet the effect on natural ecosystems, in the scope of biologically mediated transport and biomagnification of Hg and Hg-isotopes, is unknown. Using geochemistry (THg) and stable isotopes (N, Sr and Hg), we evaluate natal origin and trophic position of brown trout (*Salmo trutta fario*), as well as mercury biomagnification trends and potential pollution sources to three high-altitude lakes. Farmed trout show Hg-isotope signatures similar to marine biota whereas wild trout shows Hg-isotope signatures typical of fresh water lakes. Stocked trout initially show Hg-isotope signatures similar to marine biota. As the stocked trout age and shifts diet to a higher trophic level, THg concentrations increase and the marine Hg isotope signatures, induced via farm fish feed, shift to locally produced MeHg with lower δ^202^Hg and higher Δ^199^Hg. We conclude that stocking acts a humanly induced biovector that transfers marine Hg to freshwater ecosystems, which is seen in the Hg-isotopic signature up to five years after stocking events occurred. This points to the need of further investigations of the role of stocking in MeHg exposure to freshwater ecosystems.

## Introduction

The internationally prioritized contaminant mercury (Hg) is a potent cardiovascular- and neuro-toxic compound^1–3^ which in its organic form methylmercury (MeHg) is highly prone to both bioaccumulation and biomagnification^4^. The rapidly evolving interest in mercury’s isotopic composition have advanced the current knowledge of Hg-related processes^5^, yet many questions remain regarding source and trophic transfer of Hg in natural systems, especially those that are linked to biologically mediated transports^6^. Blais *et al*.^6^ showed that migrating birds and fish could become the predominant pathway of contaminant transport, and other studies have shown that biovector transport of Hg from ocean to rivers, by migrating salmon, constituted a substantial portion of the rivers MeHg budget^7^. Another example of biological mediated transport was shown by Senn *et al*.^8^ who concluded that transport of MeHg can occur via fish migration from costal to open ocean. Combined, these studies show that biological mediated transport of contaminants can occur both from ocean to terrestrial environment and vice versa^7–11^.

In mass-balance calculations however, marine and fresh-water food webs are often investigated separately and treated as two distinctly different ecosystems with little overlap between them. An exception to this is fish farms where fresh-water fish is raised on a high energy diet with proteins of marine origin^12^ potentially leading to elevated concentrations of contaminants^13^. As these fish are transferred back into natural freshwater systems via stocking, they become a humanly induced biovector with the potential to transfer contaminants, such as Hg and PCBs^6,7^, that can bioaccumulate and biomagnify up the food chain in the new ecosystem^13^.

Stocking lakes with non-indigenous species and farm-reared predators, such as trout, is a worldwide activity on both geographical and historical scales. With the exception of Antarctica, non-native fish can today be found in lakes and watersheds on all continents of the globe^14^. Compared with the first estimates made nearly three decades ago^15^ the number of non-native species introduced worldwide has now more than doubled^16^. Within the European Union alone, a yearly average of 132 million juvenile trout is produced for stocking purposes^17^, yet the consequences of these non-native introductions from a biogeochemical perspective, in the scope of biomagnification of Hg and Hg-isotopic signatures, is still unknown.

**Figure 1:**
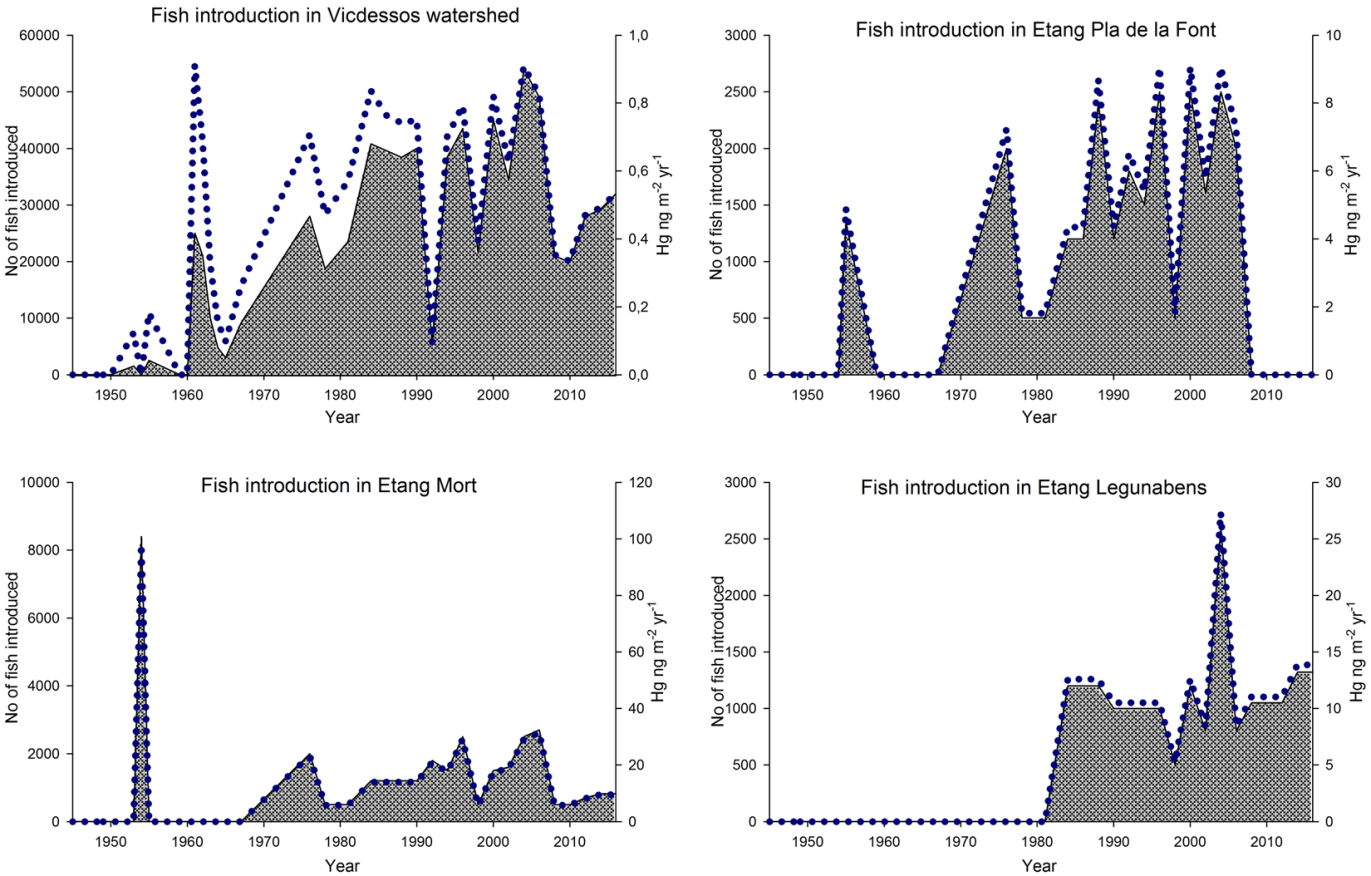
Stocking history in Vicdessos watershed. Number of brown trout introduced (grey filled area, left y-axis) to (**a**) All lakes in the Vicdessos watershed subjected to stocking, (**b**) Etang du Pla de la Font, (**c**) Etang Mort and (**d**) Etang Legunabens, and the equivalent estimated tot-Hg input in ng m^−2^ yr^−1^; where m^−2^ represents lake surface area (blue dotted line, right y-axis) vs. year (x-axis).

Historical records indicate that the introduction of brown trout (*Salmo trutta fario*) to lakes in the Ariège-region (France), where our study sites are located (Figure S1 and Table S1), commenced in the 1950s (Fig. 1a) although stocking of lakes using other species occurred already at the onset of the 20^th^ Century^13^. The first introductions were small scaled, with 1500–2500 individuals stocked in ~8 lakes, but within just a decade, the number of fry introduced had multiplied by a ten-fold. Based on historical records (Archives of the Forestry Services, France) we estimate that >80% (n = 504) of the lakes in the central French Pyrenees are, or have been subject to, stocking of farm-reared fish during the last century, and that trout constitutes 30–60% of the species being introduced. This estimate is in agreement with previous studies from the southern Pyrenees; i.e. *S. trutta* was the most widely distributed with an occurrence of ~50% in the investigated lakes^18^. Yet as the survival rate and reproduction success of these introduced trout is questionable^18–20^, 30–40 lakes in the Vicdessos watershed alone, including our three study sites, are therefore continuously stocked on a bi-annual basis since late the 1970s.

Mercury has seven stable isotopes (^196^Hg, ^198^Hg, ^199^Hg, ^200^Hg, ^201^Hg, ^202^Hg, and ^204^Hg with an abundance of 0.16%, 10%, 16.9%, 23.1%, 13.2%, 29.7% and 6.8% respectively) and as a result of fractionation during photochemical reactions, and by mixing of isotopically distinct reservoirs, the Hg-isotopic composition varies in natural samples^21^. The stable isotopes of Hg can undergo both mass-dependent (MDF) and mass-independent (MIF) fractionation. MDF is reported as δ^202^Hg (‰) and is caused by environmental processes such as methylation and demethylation^22,23^, whereas MIF is reported as Δ^199^Hg (‰) and occurs via photochemical degradation of monomethylmercury (MMHg) and/or photoreduction of inorganic mercury (IHg)^5^. Over the past decade, studies of Hg-isotopic composition in natural samples have been proven a powerful tool to trace Hg-cycling during biological processes such as bioaccumulation and biomagnification^24–26^.

Here, we use multiple stable isotopes (N, Sr, and Hg) in combination with THg to evaluate the natal origin and trophic position of brown trout (*Salmo trutta fario*) as well as mercury biomagnification trends and potential mercury sources in three high-altitude lakes in the French Pyrenees, where continuous stocking have occurred for the last 30+ years (Fig. 1 and Figure S1). Further, we also include brown trout from a local fish farm that provides the fry for all stocking activities in the region, including those at our three study sites. Our aim is to estimate the potential Hg-load that is introduced via stocking and, in comparison to regional THg atmospheric deposition, determine whether this input is significant enough to be considered a source of Hg-contamination to the freshwater aquatic ecosystem.

## Results and Discussion

### THg in farmed feed and fish

**Figure 2:**
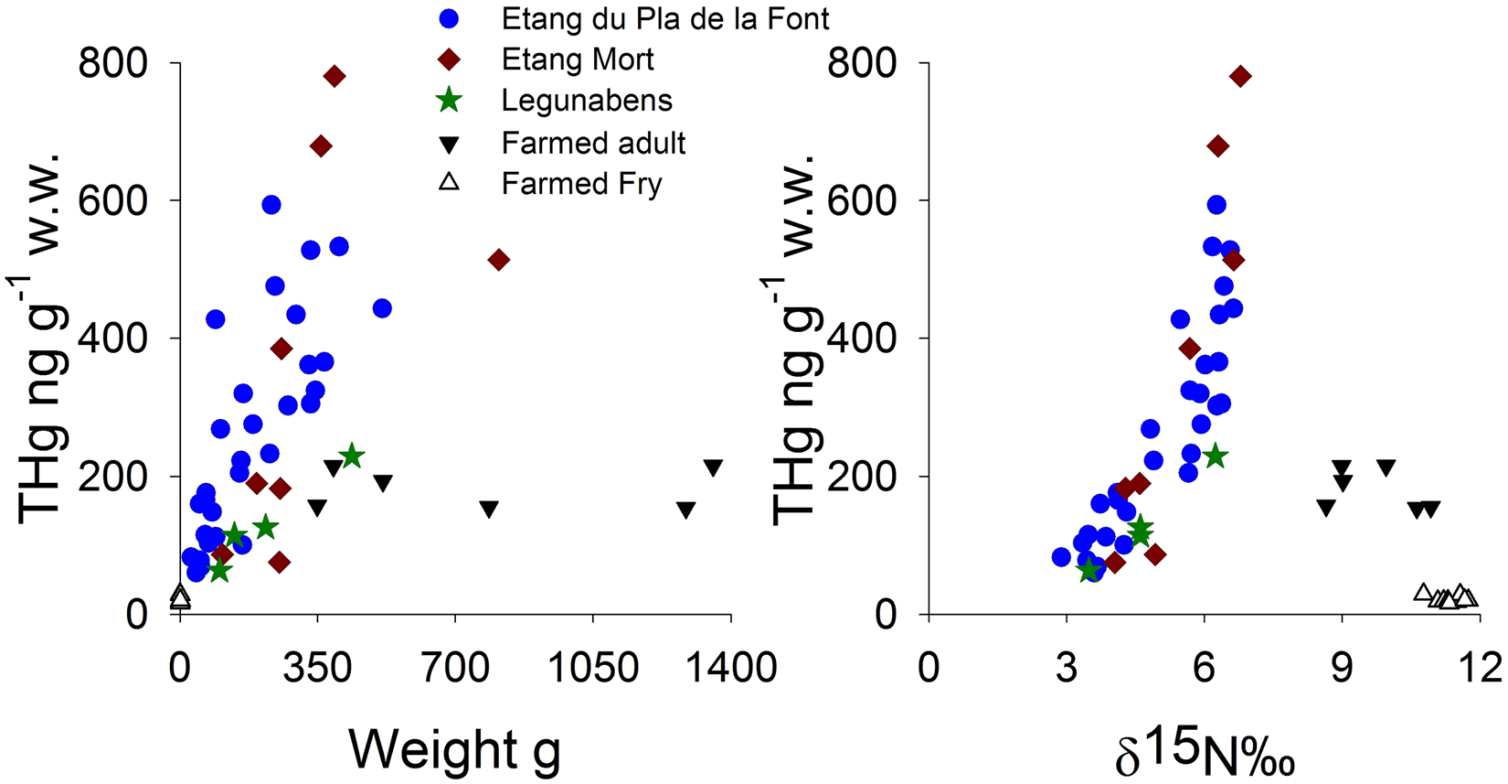
Ecological features and THg concentrations. (**A**) Concentration of tot-Hg (ng g^−1^ wet weight) in trout muscle vs weight of trout (g) and (**B**) tot-Hg (ng g^−1^ wet weight) vs δ^15^N in muscle. Blue filled circles represent trout from Etang du Pla de la Font, red filled diamonds represent trout from Etang Mort, green filled stars represents trout from Legunabens, black filled downward triangle represents farmed adult trout, and open triangle represents farmed fry.

THg concentrations in fish feed (adults and fry) ranged from 69 to 106 ng g^−1^ (mean = 77 ± 13 ng g^−1^ w.w., n = 10) for big pellets and 17 to 18 ng g^−1^ (mean = 18 ± 10 ng g^−1^ w.w., n = 10) for small pellets, and thus falls well within EUs directive (Directive 2002/32/EC On undesirable substances in animal feed) set to 200 ng g^−^^1^ for compound feed for fish. Although the concentrations of THg in the feed may have varied over time due to differences related to production and/or producers^27^, the concentrations seen in our farmed samples should be considered a representative value as similar concentrations of Hg have been shown in previous studies on fish feed, i.e. THg concentrations ranging from 9 to 90 ng g^−1^ ^13,28^^)^. This is further supported by the fact that all adult farmed trout, age 3 and 4 yr, which have been entirely raised on these marine derived protein diet^12^, shows maximum THg values of ~200 ng g^−1^ w.w. (Fig. 2), and therefore do not exceed the 500 ng g^−1^ THg w.w. limit set by the EUs Commission regulation on maximum levels for certain contaminants in foodstuffs (EC NO1881/2006). Although our study is based on only 16 brown trout from one fish farm (in addition to the 42 fish caught at our study sites), all samples from the farm falls well within regulatory values and data reported elsewhere^12,28^, thus allowing us to further discuss the fish feed – stocking – freshwater ecology interplay.

### Natal origin of fish – Otoliths and Sr-isotopes

**Figure 3:**
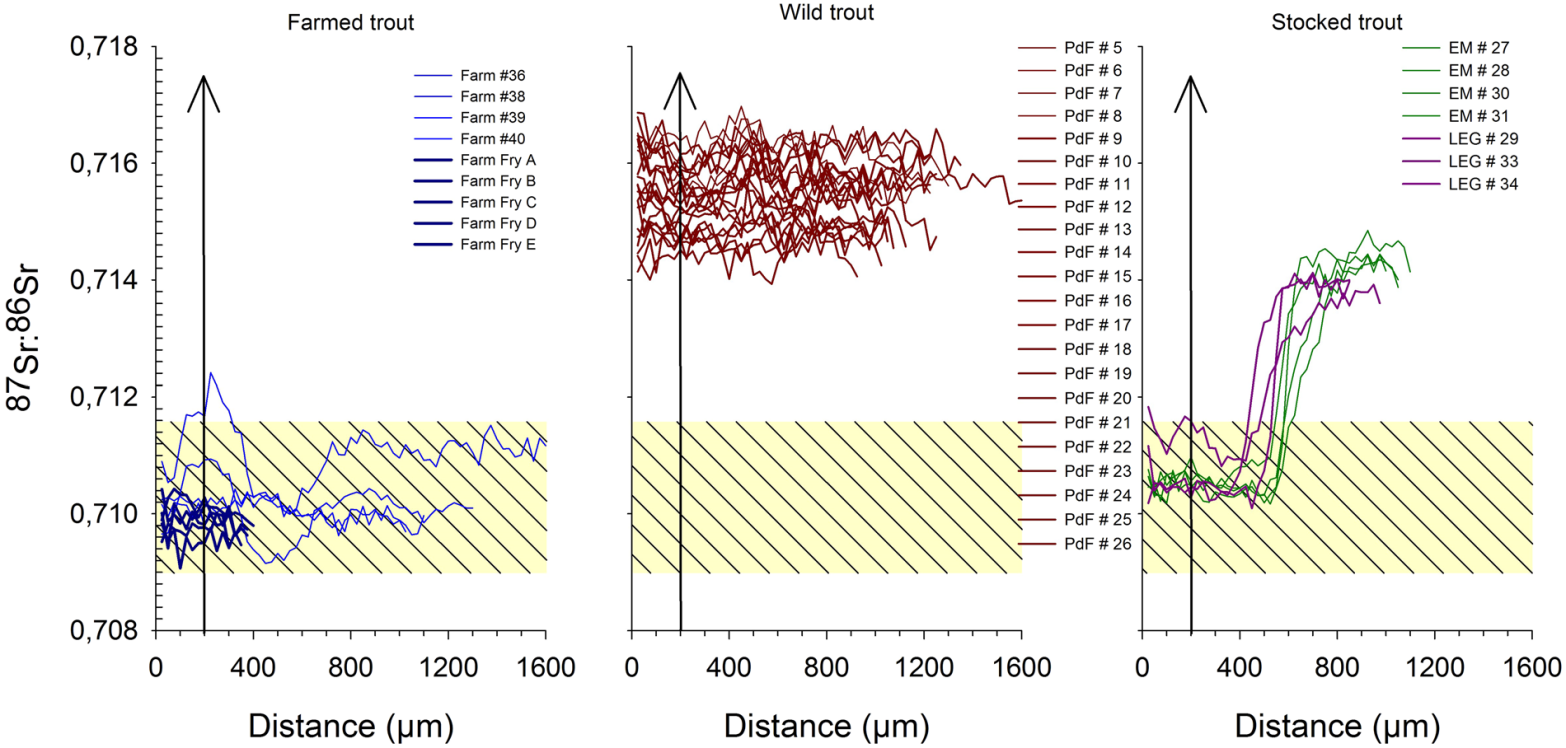
Natal origin of each individual fish. ^87^Sr/^86^Sr isotopic composition in individual otoliths, presented as ‰, vs distance in µm from the center of the otolith as measured in farmed trout (left), wild trout (center) and stocked trout (right). Each line represents one individual fish and is color-coded with site of collection; fish farm (blue), Etang du Pla de la Font (red), Etang Mort (green) and Legunabens (purple). Note that for the fish-farm fry and adult trout are displayed in dark-blue (fry) or blue (adult). For visual aid, the ^87^Sr/^86^Sr isotopic composition in the farmed trout are presented as yellow box with black stripes in all three graphs.

To distinguish natal origins and movement of fish stocks collected from our three sites (Fig. 3 and Table S1 Supplementary information), we here use ^87^Sr/^86^Sr isotope ratios in fish otoliths^29–31^ and compare these to otoliths in farmed fish. As ^87^Sr/^86^Sr isotope ratios are incorporated unchanged in freshwater fauna, i.e. with no biological fractionation and no temperature effects^32,33^, the Sr isotopic composition in otoliths of wild trout directly reflects the surrounding water in which the fish resides, i.e. the lake water chemistry^32^. The Sr-isotopic ratio recorded in farmed trout however should be a mixture between hatchery water chemistry and fish feed as the latter would not be in equilibrium with Sr isotopic composition of hatchery water^32^. The presence of crystalline underlying geological substrate (with high expected ^87^Sr/^86^Sr ratios^33^) could help discriminate fish that were born in our studied lakes from hatchery-reared fish that have experienced water draining sedimentary environments^34^. Based on^87^Sr/^86^Sr isotope profiles in otoliths of selected trout caught at our study sites, and trout purchased at a local fish farm, we can distinguish individual fish born in the wild to those that have been stocked^31^. As seen in Fig. 3, the isotopic signatures showed distinct differences with an ^87^Sr/^86^Sr ratio of 0.7090–0.711 ± 0.0005‰ (Fig. 3a) for the farmed fish and 0.7140–0.7165 ± 0.0005‰ (Fig. 3b) for the wild fish. Although variable among individuals, the ^87^Sr/^86^Sr-ratio stays relatively constant during the entire life of the individual fish of wild origin^35^, thus allowing us to unambiguously differentiate wild from stocked trout (Fig. 3c). Stocked trout shows a drastic increase in ^87^Sr/^86^Sr-ratio from 0.7102–0.7111‰ at the inner section of the otolith (corresponding to maternal influence and early life stages, and also falling well within the range of our farmed trout), to a ^87^Sr/^86^Sr-ratio of 0.7132–0.7147‰ at the time of death, corresponding to the ^87^Sr/^86^Sr-ratio seen in our wild fish. This switch in ^87^Sr/^86^Sr-ratio is not instantaneous, i.e. studies have shown that the “new” Sr isotopic composition will be evident within three months of stocking^32^ and some time lag may therefore occur before the ^87^Sr/^86^Sr-ratio incorporated in the otolith fully reflects that of the new environment. However, as all trout selected for ^87^Sr/^86^Sr-isotopic analysis has a minimum age of 1 yr, thus yielding sufficient time to incorporate a clear chemical signature, the switch in isotopic composition as seen in Fig. 3c must therefore represent farm-reared trout that have been stocked into the lakes at young age.

The results of ^87^Sr/^86^Sr-ratio in otoliths (Fig. 3) shows that there are no wild trout in neither Etang Mort nor in Etang Legunabens. This is further supported by historical records claiming that as neither of these two lakes are able to sustain a natural reproduction of brown trout (*Salmo trutta*), both lakes are continuously stocked on a bi-annual basis since the mid-1970s (Etang Mort, Fig. 1c) and mid-1980s (Etang Legunabens, Fig. 1d; Archives of the Forestry Services, France). The opposite situation is seen in Etang du Pla de la Font, where the ^87^Sr/^86^Sr-ratio in selected trout otoliths show no farm-reared ^87^Sr/^86^Sr-signature, and that all trout from Etang du Pla de la Font subjected to ^87^Sr/^86^Sr –analysis must be of wild origin. This is again in good agreement with the historical records as the last introduction of farmed fry to this lake occurred in 2006 (Fig. 1b). As we have no trout older than 5 years from this lake in our dataset, any trout younger than 8–9 years at the time of our sampling (2014 and 2015) must be the product of natural reproduction. When compared to wild fish, stocked farm-reared fish show reduced feeding^36^ and territorial efficiency^37^, as well as a higher mortality rate^38^. As it has been shown that the growth rate of wild native trout is unaffected by stocking of farm-reared trout^39^, and since there already exist a wild population of brown trout in Etang du Pla de la Font, wild trout is expected to be the most abundant.

### Hg-isotopes (δ^202^Hg Δ^199^Hg) in farmed fish

**Figure 4:**
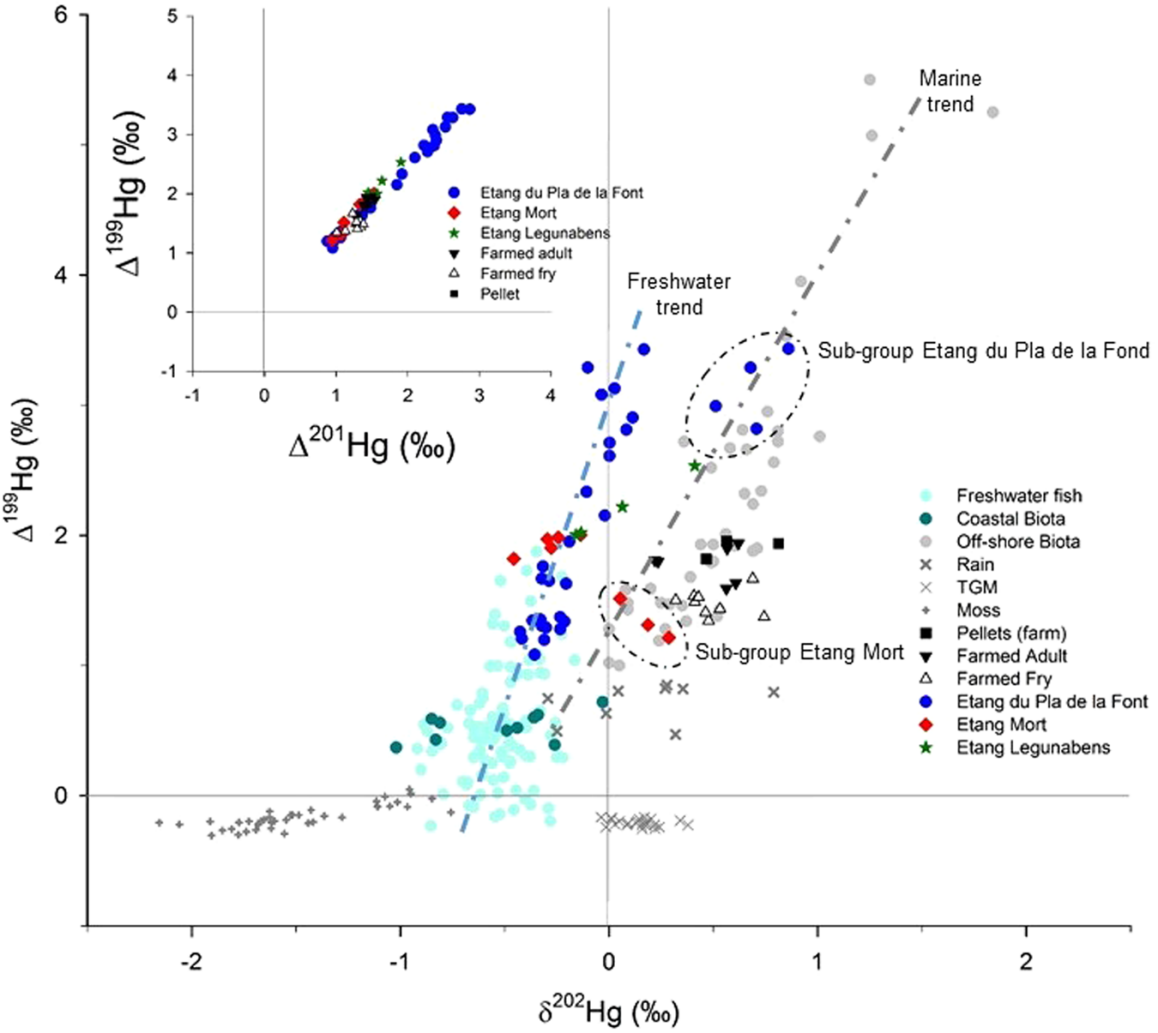
Hg-isotopic composition in studied vs literature samples. δ^202^Hg vs Δ^199^Hg in trout muscle from Etang du Pla de la Font (blue filled circle), Etang Mort (red filled diamond) and Legunabens (green filled stars) in Bassiès valley, as well in muscle from adult farmed trout (black filled downward triangle), farmed fry (open triangle) and pellets (black filled square). Two sub-groups, i.e. in Etang du Pla de la Font and in Etang Mort, are displayed with a black dashed-line ellipse. Literature data on isotopic Hg-composition are shown for comparison, i.e. data on off-shore biota (grey filled circles^31,32^), costal-biota (dark cyan filled circles^31^), freshwater fish (light cyan filled circles^35,36^), TGM (dark grey thin cross^39^), rain (dark grey thick cross^16^), and *Sphagnum* moss data (dark grey plus^16^). Data on Δ^201^Hg vs Δ^199^Hg in fish muscle from Bassiès and fish farm is shown as superimposed graph in the upper left corner.

The characterization of wild versus stocked origin, based on Sr-isotopic signatures, is essential to understanding the THg concentrations (Fig. 2) seen in the trout but also to decipher the potential sources of Hg, using Hg isotopes, in the three high mountain lakes (Figs 2 and 4). As shown in Fig. 4, the three categories of origin; farmed, stocked or wild, as evidenced by the ^87^Sr/^86^Sr-signatures in fish otoliths, is also reflected in the Hg-isotope signatures:

Farmed fish, both adults, fry and pellets, exhibits a total variation of δ^202^Hg from 0.22 to 0.81‰ and in Δ^199^Hg from 1.34 to 1.95‰, corresponding well to data from off-shore biota (average δ^202^Hg = 0.41‰ and Δ^199^Hg = 1.75‰) as reported by Senn *et al*.^8^ (Fig. 4). For example, Blum *et al*.^40^ reported an average Hg isotopic composition in Opah (moonfish) to be δ^202^Hg = 0.54‰ and Δ^199^Hg = 1.87‰, and δ^202^Hg = 0.57‰ and Δ^199^Hg = 1.88‰ in Broadbill swordfish, thus comparable to both our farmed adult trout and farmed fry displaying an average of δ^202^Hg = 0.47‰ and Δ^199^Hg = 1.78‰, and δ^202^Hg = 0.50‰ and Δ^199^Hg = 1.48‰ respectively. This signature of marine biota is also reflected in the pellets (δ^202^Hg = 0.50‰ and Δ^199^Hg = 1.48‰) corresponding to data from Blackfin Tuna (δ^202^Hg = 0.37‰; Δ^199^Hg = 1.49‰) as reported by Senn *et al*.^8^, and in agreement with studies showing that the majority of farmed fish in the European Union is raised on fish meal of marine origin as main source of protein^12^. This is further confirmed by historical records stating that since the 1960s, the main source of protein in the food fed to farmed fish in France is based on fishery products of marine origin. As diet is the dominant pathway of Hg uptake by fish^41^, and fish assimilate the dietary δ^202^Hg and Δ^199^Hg signatures within just 10 days^25^, a marine δ^202^Hg and Δ^199^Hg-signature as seen in our farmed fish is therefore expected.

Earlier studies have shown that Hg isotopic composition varies in oceanic, coastal and terrestrial biota, which is caused by different sources of Hg species, and (or) different reaction pathways^8,26,40^. The δ^202^Hg value therefore varies between marine and freshwater fish as they are subjected to different IHg sources that undergo methylation before entering the food chain. Marine areas receive IHg mainly from atmospheric IHg(II) wet deposition, whereas freshwater ecosystems receives Hg from the surrounding terrestrial watershed^42–44^. Terrestrial watersheds in turn receive IHg predominantly from plant uptake of atmospheric gaseous elemental Hg(0) (GEM). Since Hg (II) wet deposition and Hg(0) plant uptake have contrasting δ^202^Hg Δ^199^Hg signatures, the IHg isotope baseline in freshwater and marine ecosystems is different, leading to distinguishable MeHg δ^202^Hg and Δ^199^Hg^45–48^.

### Hg-isotopes (δ^202^Hg Δ^199^Hg) in wild fish

In contrast to the marine Hg-isotopic signature seen in the farmed fish with positive δ^202^Hg values, the wild trout from Etang du Pla de la Font (Fig. 4), instead show a δ^202^Hg variation of −0.43 to 0.86‰ (mean ± σ = −0.024 ± 0.37‰) and Δ^199^Hg variation of 1.08 to 4.56‰ (mean ± σ = 2.26 ± 0.96‰). These values corresponds well to Hg-isotopic composition of fresh-water fish reported elsewhere^5,24,49–51^. The positive fish Δ^200^Hg-signature (−0.02 to 0.18‰, mean = 0.10 ± 0.04‰; 0.050 to 0.18‰, mean = 0.11 ± 0.03‰; 0.08 to 0.15‰, mean = 0.11 ± 0.04‰ for Etang du Pla de la Font, Etang Mort and Etang Legunabens respectively) in the wild trout are characteristic of a mixture of Hg(II) wet deposition and plant Hg(0) uptake. Low fish δ^202^Hg are compatible with the important contribution of plant Hg(0) to the watershed, while elevated Δ^199^Hg likely reflects in-lake photochemical breakdown of MeHg (see Table S3 for detailed information on Δ^200^Hg values in all samples, including the farmed fish and fry).

Previous studies have shown relationships between Δ^199^Hg and δ^15^N in freshwater food chains^52,53^, i.e. a significant increase of Δ^199^Hg with each higher trophic level, stating that some *in vivo* MIF in fish may in fact occur. It has been argued however, on both theoretical^54^ and experimental^24,25^ grounds, that *in vivo* metabolic processes are not likely to cause MIF of Hg isotopes, and that MeHg will undergo bioaccumulation and biomagnification without MIF^8,55^. Although we also see a correlation between Δ^199^Hg and δ^15^N in our lakes (Figure S2), the high Δ^199^Hg as seen in some of our trout is likely caused by photoreduction of MeHg. As also shown by Li *et al*.^56^, the Δ^199^Hg/Δ^201^Hg-isotopic trend in our trout (Fig. 4) varies from 1.24 to 1.32 (Δ^199^Hg/Δ^201^Hg = 1.24, 1.32 and 1.30 for Etang du Pla de la Font, Etang Mort and Etang Legunabens respectively) and are in better agreement with photochemical demethylation of MeHg (1.36 ± 0.02, 2SE) than with photoreduction of Hg(II) (1.00 ± 0.02, 2 SE)^5^, and therefore reflects demethylation processes of MeHg in the lake and the mountain streams prior to uptake and incorporation in the lake food web^5,8,40,51,57^.

### Hg-isotopes (δ^202^Hg Δ^199^Hg) in stocked fish

The marine signature, with positive δ^202^Hg and relatively low Δ^199^Hg as seen in our farmed fish, is not found in the wild trout in Etang du Pla de la Font. Yet one sub-group, collected in or near the stream inlet of the lake, displays positive δ^202^Hg and high Δ^199^Hg values, thus overlapping in part with the marine trend (δ^202^Hg variation of 0.51 to 0.86‰, mean = 0.69 ± 0.14‰; and Δ^199^Hg variation of 2.82 to 4.56‰, mean = 3.13 ± 0.28‰) seen in literature^8,51^. In contrast to public records, stating that no fish has been introduced in Pla de la Font since 2006, it is our interpretation that these fish must originate from the fish farm as they also show lower THg and δ^15^N values (see discussion regarding sub-group in Etang Mort below). However, as no ^86^Sr/^87^Sr-analysis were made on these particular individuals (Sr-analysis were performed only on fish collected in 2014) we can only speculate on the origin and thus also the isotopic composition of these fish. Other potential explanations could be linked to a difference in Δ^199^Hg/δ^202^Hg slope associated with the photoreduction of MeHg between the stream and the lake due to variation in light exposure^58^. A higher δ^202^Hg of stream IHg or MeHg due to local stream-level variations in Hg deposition sources (i.e. more rainfall IHg(II)) or methylation conditions can technically also lead to the high fish δ^202^Hg. We consider however, that undocumented stocking is the most likely explanation for the observed marine δ^202^Hg in Etang du Pla de la Font.

In contrast to Etang du Pla de la Font, the marine signature seen in the farmed fish can also be seen in the Hg-isotopic composition of the stocked trout in Etang Mort with a δ^202^Hg variation of −0.46 to 0.29‰, mean = −0.11 ± 0.26‰ and Δ^199^Hg variation of 1.21 to 2.00‰, mean = 1.72 ± 0.32‰. Again two sub-groups can been seen within the same lake; one (n = 3) showing Hg-isotopic signatures very similar to that of the farmed fish, whereas the other (n = 5) appears to have shifted to similar Hg-isotopic signatures as seen in the wild trout of Etang du Pla de la Font. Although there is no correlation with age (Figure S2), the sub-group that has maintained their marine-signature has a slightly lower δ^15^N (mean = 4.46 ± 0.38‰) and lower THg (mean = 134 ± 62 ng g^−1^ w.w.) versus that which has shifted towards the wild signature (mean δ^15^N = 6.35 ± 0.49‰; mean THg = 589 ± 175 ng g^−1^ w.w.). It therefore appears as part of the trout population in Etang Mort, displaying lower Δ^199^Hg and higher δ^202^Hg, feeds from a lower level within the food chain, and by doing so also maintains the marine Hg-isotopic composition for a longer time period (age 3–5 years) than the part of the population that has shifted their diet towards a higher trophic level (age 4–7).

This “ability” to maintain the marine Hg-isotopic signature can in part be explained by the long half-life of MeHg in fish (1–4 years^56^) but more importantly, by the overall THg concentration in the new diet. Previous feeding experiments^24,25^ have shown that when subjected to a new diet containing a different Hg isotopic composition, the Hg-isotopic signature in fish will shift to values close to that of the new diet within 10 days and fully equal the Hg-isotopic signature of the new food source within 30 days. These results however are based on experiments where the fish was subject to a dietary shift going from a natural low THg diet to a high THg experimental diet^25^. However, when fish was subjected to a low THg-diet, the Hg-isotopic shift was incomplete and did not fully reflect the new diet at the end of their 80 days study. The lower δ^15^N and THg concentrations, as seen in some of the stocked trout in Etang Mort, would indicate that these trout have not yet bioaccumulated enough local MeHg to shift the Hg-isotopic composition in their muscle tissue.

As trout are an opportunistic species^59^, with known cases of cannibalism^60^, another potential explanation for persistent marine Hg-isotopic signatures seen in Etang Mort could be that the biannually introduced fry becomes an easy prey for adult trout, thus allowing the stocked trout to maintain the marine rearing Hg as a dominant THg source. This should however also yield higher δ^15^N values^61^ due to the 2 to 3 fold higher δ^15^N-signature (11.37‰ in fry vs 4.46 to 6.36‰ in Etang Mort; Fig. 2) shown in the farmed fry, and that cannibalism would indicate a dietary shift towards larger prey^62^. Yet as the sub-group that has maintained the marine δ^202^Hg signature has a lower δ^15^N (mean = 4.46 ± 0.38‰) than the group displaying a Hg-isotopic signature of atmospheric origin (mean = 6.35 ± 0.49‰), cannibalism cannot explain the δ^202^Hg off-set between the two sub-groups, but rather the lack of dietary shift^59^ and slow turn-over of tissue signatures due to a slower growth rate^63^, i.e. 54 g yr^−1^ for the sub-group with marine signature vs 96 g yr^1^ for the sub-group with atmospheric signature.

Based on the Hg-isotopic composition (δ^202^Hg variation of −0.16 to 0.41‰ and Δ^199^Hg variation of 2.00 to 2.43‰; Fig. 4), the trout in Etang Legunabens appears to fall intermediate between that of Pla du Font and Etang Mort. Although less clear as in Etang Mort, the same sub-groups based on δ^15^N (~4 and ~6; Fig. 2) seem to exist, yet opposite to Etang Mort, the higher δ^15^N value also means higher δ^202^Hg and higher Δ^199^Hg, i.e. more similar to the second sub-group seen in Etang du Pla de la Font. In contrast to Etang Mort, and to some extent Etang du Pla de la Font, Etang Legunabens has no peatland in its direct vicinity, indicating a lower degree of DOC and MeHg input^64,65^. This would yield an increased depth of light penetration in the water column resulting in more intense photochemical demethylation of MeHg, and would explain the higher Δ^199^Hg than Etang Mort^58,66^. It would also explain the lower Δ^199^Hg than Etang du Pla de la Font as all trout in Etang Legunabens were caught in the lake, i.e. in the limnetic to profundal zone, and not the littoral and riverine zone, where the wild trout displayed overall highest values of both δ^202^Hg and Δ^199^Hg.

### Estimated introduction of THg and potential marine-terrestrial MeHg transfer

A recent inventory by Kocman *et al*.^67^ estimates that 800–2200 Mg Hg yr^−1^ is released to freshwater ecosystems from anthropogenic sources on a global scale. To our knowledge, no study have previously looked at the Hg released to freshwater systems via stocking as a local Hg source.

Based on historical records of fish introduction, lake volume and THg concentrations as measured in trout purchased at a local fish farm (providing the fry used when stocking lakes in the region), we estimate that an average of 14 ng THg m^−2^ yr^−1^ (equivalent to 8 ng THg m^−3^ yr^−1^, with metric units referring to lake surface and volume) is introduced to the three lakes in the valley of Bassiès on a yearly basis (Fig. 1b–d). Compared to an average regional atmospheric wet deposition of 9.3 µg THg m^−2^ yr^−1^ ^45^, as measured on a peatland 50 km from our study sites, farm-reared Hg introduced via stocking does not appear as a significant pollution source. This is confirmed by the loss of the marine Hg isotope signature as the fish grows. However, it is important to keep in mind that the 14 ng THg m^−2^ yr^−1^ introduced via stocking is directly bioavailable MeHg to predator fish at higher trophic levels, as 85–95% of the THg in fish is in the form of MeHg^68^. For instance, previous studies^7^ have shown that biological Hg transport of migrating wild salmon in Alaska introduced up to 1 kg yr^−1^ MeHg, which constituted a significant portion of the rivers MeHg budget. It would therefore be more accurate to compare the input of MeHg via stocking to input of MeHg via regional atmospheric deposition.

To establish a fully detailed MeHg-budget is beyond the scope of our study, but for the benefit of our discussion we conducted a simplified comparison of MeHg concentrations in wet deposition, surface sediment and stocked fish during the year 2014 (time of our sampling campaign). We chose Etang Legunabens as an example due to its intermediate size, relatively isolated location with only one small stream as an inlet, and the absence of any peatland within its catchment^69^.

In 2014, an estimated total of 560 µg MeHg (see Methods-section for further details) were introduced to Legunabens via direct wet deposition whereas 178 µg MeHg were introduced via farmed trout. In addition, the top 0–2 cm sediment layer showed an estimated MeHg concentration of 0.004 µg g^−1^ and combined, this would give a total of ~738 µg MeHg in Legunabens in 2014 (excluding any surface runoff or additional input from the surrounding catchment). Out of the total 738 µg MeHg in Legunabens, 24% would thus be introduced via stocked trout. We stress that this is a rough estimation calculated based on broad assumptions, yet it clearly shows that although Hg introduced via stocking may be a small source it is not insignificant in the local freshwater ecosystem.

If we further extrapolate the THg-concentrations from our farmed fish on a global scale, assuming a worldwide yearly production of 2.6 million ton for inland aquacultures and freshwater salmonids alone^70^, aquacultures would represent a potential net-transfer of 0.1 Mg THg per year of marine Hg to the continental environment, of which 85–95% would be in the form of MeHg^68^. Considering all inland aquacultures and freshwater species, i.e. 29 million ton production per year^71^, and assuming a bodily Hg concentration similar to that measured in the farmed trout (38 ng g^−1^ at a weight of 3.5 to 4 g), the potential net-transfer of marine MeHg to continental freshwater ecosystems is ~1 Mg per year. Compared to a 500–1260 Mg Hg yr^−1^ released to freshwater ecosystems from ASGM^67^, or a 5500 ± 2700 Mg Hg yr^−1^ discharge from river to ocean^42^, this humanly induced biovector transport of marine Hg via stocking is thus still small.

It should be noted that as our estimate is based on just 16 samples from one fish farm, further studies including more fish farms and stocked lakes, as well as more detailed data from grey literature, is needed in order to draw robust conclusions on the importance of stocking in global mass-balance calculations. However, the fact that we still see a dominant marine Hg-isotopic signature in some of our fish up to five years after stocking indicates that the marine (Me)Hg affects the local aquatic mercury cycle and that there is a need for further studies on stocking as a potential MeHg source to freshwater ecosystems.

## Conclusion

Based on all our data combined, we conclude that introduction of farmed brown trout (*Salmo trutta fario*) to our three mountain lakes act as a small source of THg to these high-altitude aquatic ecosystems, with an estimated average input of 14 ng m^−2^ yr^−1^ MeHg. It is also clear that the pellets used when raising fry at fish farms, based on protein from fishery products of marine origin^12^, will render Hg-isotopic signals in both farmed fry and adults comparable to that of top-predator marine biota as reported in literature^8,40^. Depending on diet, growth rate and trophic position, this marine-reared isotopic signature can still be seen in the adult trout up to 5 years after introduction to natural freshwater ecosystems. Stocking of farmed fish into freshwater ecosystems therefore act as a humanly induced biovector, potentially transporting up to 1 ton of marine MeHg per year to continental areas, yet further studies are needed to confirm this value.

## Methods

### Site description and history of stocking

All study sites, Etang du Pla de la Font (42°45′52.79″N, 001°25′09.77″E, 1653 m a.s.l.; 2.9 ha) Etang Mort (42°45′52.77″N, 001°25′28.86″E, 1676 m a.s.l.; 0.9 ha), and Etang Legunabens (42°45′52.85″N, 001°25′52.25″E, 1675 m a.s.l.; 1.0 ha) are located in the Bassiès valley, Vicdessos, approximately 150 km south of Toulouse, France (Figure S1). Although all three lakes are located on the same Bassiès granitic batholith^72^ bedrock, and within a one km proximity of each other, their immediate surrounding varies between each site. Etang du Pla de la Font is situated with *Sphagnum* dominated peat to the West-North-East, and rock formations to the East-South-West. Etang Mort is directly surrounded by a *Sphagnum* dominated peatbog on all sides with the exception of a few rock formations at the south/south-west side part of the lake. Legunabens is, on the contrary, surrounded by steep facing rock-formations, thin soil covers and scarce vegetation dominated by heathlands (*Calluna vulgaris*) and shrubs (*Rhododendron ferrugineum*).

Although some early fish introductions were carried out until the 18^th^ century in the Etang du Pla de la Font for commercial purpose^73,74^, the continuous stocking of brown trout (*Salmo trutta fario*) to all three lakes started in the mid-1970s (Archives of the forestry services, France). Since then an estimated total of 79,000 fry, all rearing from the same local fish farm, have been introduced. Yet successful reproduction and establishment of a “wild” (or “neo-native”) population has only occurred in Etang du Pla de la Font where natural reproduction is possible due to the presence of large streams, whereas all trout in both Etang Mort and Etang Legunabens are stocked.

### Sampling and sample preparation

Samples of brown trout (*Salmo trutta fario*) were collected in October 2014 and October 2015 using either electric (Etang du Pla de la Font) or net (Etang Mort and Etang Legunabens) fishing. Upon capture all fish were sacrificed with an overdose of anesthetic solution (120 mg/L Benzocaïne), following local guidelines and regulations. Length and weight of each fish was recorded (Table S1), and scales were collected for age determination following the protocol by Schneider *et al*.^75^. The fish were then rapidly transported at 4 °C (mixture of ice and dry ice) to the lab and dissected following the protocol by ICP Waters report 105/2010. Muscles and heads were frozen after dissection and maintained at −20 °C until further analysis. Trout purchased at the local fish farm were subjected to the same procedure (sacrificed and dissected) as the trout caught at our study sites. The only exception to the dissection procedure stated above was the 10 fry samples from the fish farm. Due to their limited size, dissection was deemed too difficult, and the samples where therefore frozen intact and treated as bulk of that individual.

All samples, with the exception of fish heads, were lyophilized using a Christ Alpha 1–2 freeze drier, and then homogenized by manually grinding each sample into a powder using an acid cleaned agate mortar, then placed in falcon tubes and stored under dark and cool conditions until further analysis.

Approximately 100 mg of dried and homogenized material was placed in acid cleaned digitubes (SCP Sciences 010–500–263) together with 3 mL of nitric acid (HNO_3_; ~67–69%), closed with airtight caps, and digested overnight at 90 °C. After being cooled down to room temperature the digestions were diluted to reach a final THg concentration of 1 ng g^−1^ in 20 vol% acid by dilution with mQ-H_2_O and 20% aqua regia. All sample preparations were performed under clean laboratory conditions using acid cleaned lab ware.

### Otoliths and fish origin

Fish heads were thawed and otolith pairs were extracted from each trout using cleaned plastic clamp, after which they were cleaned with ultrapure water and air-dried under laminar flow hood. Otoliths were then embedded in epoxy resin (Araldite 2020, Escil) in the sagittal plane, sanded to the primordium with sandpaper (1200–4000 grit) and polished, rinsed and dried before being stored in individual polypropylene vials until analysis. Analysis of ^87^Sr/^86^Sr isotopic ratios was performed at the University of Pau (France), the IPREM (Institute of analytical sciences and physico-chemistry of environment and materials), using a multicollector inductively-coupled plasma mass spectrometer (Nu-Plasma MC-ICP-MS) coupled to a UV high-repetition-rate femtosecond laser ablation (fs-LA Lambda 3,Nexeya SA, Canejan, France) system. Linear raster scans were made from 200 µm distance before the primordium to the edge of the otolith following the method outlined by Martin *et al*.^35^. Analytical accuracy was achieved through the repeated analysis of the marine fish otolith Certified Reference Material NIES 22 (National Institute for Environmental Studies, Japan^58^) during each LA run. An average ^87^Sr/^86^Sr (*n* = 16) of CRM NIES 22 of 0.70926 ± 0.00012 (2 SD) was obtained, which is consistent with the nearly constant modern seawater (0.709176 ± 0.000003^76^).

Out of the 45 trout collected from our study sites, and the 16 trout purchased at the fish farm, 42 individuals were analyzed for ^87^Sr/^86^Sr-ratio of which 40 yielded reliable results; two samples from the farmed trout were deemed unusable due to the presence of vaterite crystal on the otoliths. Yet as the origin and age of these two individuals were already known, they were still included in the Hg-isotope analysis and discussion. The remaining 17 samples were collected in a secondary field campaign in 2015, thus never subjected to otolith analysis. However, based on their δ^13^C and δ^15^N isotopic composition (Figure S2), and the known history of fish introduction, we were still able to estimate the origin of these 17 individuals, i.e. place them in either the “wild” or “stocked” group.

### THg (DMA) and δ^15^N

Total Hg concentrations were measured by atomic absorption after combustion and gold trap pre-concentration using a Milestone DMA-80 at the Midi-Pyrenees Observatory/GET Laboratory, Toulouse, France. A calibration curve was created using MESS-3 (2–6 ng), TORT-3 (10–20 ng) BCR482 (40 ng), IAEA86 (55 ng) and IAEA436 (90–350 ng) and the analytical settings was set to 300 °C and 120 sec drying phase, 850 °C and 150 sec decomposition phase with a 60 sec waiting time. To ensure the analytical quality throughout the analysis, replicates and SRMs (TORT-3 = 291 ± 45 ng Hg g^−1^, n = 40; CE464 = 4569 ± 147 ng Hg g^−1^, n = 6; and IAEA 436 = 4429 ± 692 ng Hg g^−1^, n = 16) were included after every 10^th^ samples and blanks were run after every 3^rd^ sample. The relative deviation for replicates was within 18% (average = 2.57%), recoveries were 100%, 87% and 106% for TORT-3, CE464 and IAEA respectively, and the blanks ranged from 0.01 to 0.84 ng g^−1^ (average = 0.12 ng g^−1^).

Total-carbon and total-nitrogen contents were determined using an IRMS elemental analyzer (Isoprime 100) at the SHIVA analytical platform of EcoLab Toulouse, France. Analytical quality was controlled using internal standards and replicates; Acet (n = 14) mean N = −3.47‰, 1 s.d. = 0.15 and mean C = −33.14‰, 1 s.d. = 0.04; Ala (n = 14) mean N = 8.61‰, 1 s.d. = 0.11 and mean C = −22.88‰, 1 s.d. = 0.04; and Uree (n = 19) mean N = −0.35‰, 1 s.d. = 0.05 and mean C = −35.93‰, 1 s.d. = 0.14.

### Hg stable isotopes

All sample solutions were adjusted to an acid concentration of 20% (v/v) and THg concentration of either 0.5 ng g^−1^ (fish fry and pellets) or 1 ng g^−1^ (all other samples) before Hg isotope analysis. The solutions were then analyzed for Hg isotopic ratios by cold vapor-multicollector inductively coupled plasma mass spectrometry (CV-MC-ICPMS) using a Thermo-Finnigan Neptune analyzer at Midi-Pyrenees Observatory/GET Laboratory, Toulouse, France. The international standard NIST SRM 3133 was used for mass bias correction of the isotopic ratios by sample bracketing. The results are expressed in per mil (‰) and are reported as δ-values, representing deviation from the bracketing standard;

1 $${\delta }^{xxx}Hg=(\frac{{(\frac{{}^{xxx}Hg}{{}^{198}Hg})}_{Sample}}{{(\frac{{}^{xxx}Hg}{{}^{198}Hg})}_{SRM3133}}-1)\times 1000$$

The deviation of δ-values from the theoretical Mass dependent fractionation (MDF) is quantified as mass independent fractionation (MIF);

2 $${{\rm{\Delta }}}^{xxx}Hg={\delta }^{xxx}H{g}_{Sample}-\beta \times {\delta }^{202}H{g}_{Sample}$$

where, according to the kinetic MDF law, the β-values are 0.252, 0.502, 0.752, and 1.493 for isotopes ^199^Hg, ^200^Hg, ^201^Hg, and ^204^Hg respectively. Analytical quality and reproducibility was assessed by including samples of ETH-Fluka and UM-Almaden into our measurements. ETH-Fluka (n = 9) displayed δ^202^Hg mean values of −1.47‰ (2 s.d. = 0.27‰), Δ^199^Hg mean as 0.08‰ (2 s.d. = 0.09‰) and Δ^201^Hg mean as 0.01‰ (2 s.d. = 0.06‰), and UM-Almaden (n = 6) displayed δ^202^Hg mean values of −0.49‰ (2 s.d. = 0.12‰), Δ^199^Hg mean as 0.004‰ (2 s.d. = 0.10‰) and Δ^201^Hg mean as −0.02‰ (2 s.d. = 0.07‰). Further information can be seen in Table S3 in the supplementary information.

### Estimate of Hg input vs atmospheric deposition

To verify the importance of stocking as a biovector transport of marine THg to freshwater ecosystems, we estimated the THg m^−2^ yr^−1^ input using the following equation;

3 $$Hg\,input=\frac{{\rm{Conc}}\,\,{\rm{Hg}}\,{\rm{ng}}\,{{\rm{g}}}^{-1}{\rm{w}}.{\rm{w}}.\,\,\ast \mathrm{Weight}\,\,\ast \mathrm{No}\,\,{\rm{of}}\,{\rm{fish}}}{{\rm{Area}}}$$

where Conc Hg is the estimated concentration of THg in fry at the time of introduction (38 ng g^−1^ w.w. at 3.5 g based on a regression calculation using weight vs THg as measured in farmed fish; i.e. y = 0.1454x + 37.853 R^2^ = 0,6644 where y is bodily concentration of Hg in ng g^−1^ w. w. and x is weight in g), Weight is the average weight of trout introduced based on information from historical records (3.5 g), No of fish is the annual number of brown trout introduced (based on historical records) and Area is the total lake surface area. This calculation was performed for each lake individually, and enabled us to compare the estimate THg input from stocking in relation to input from deposition. A similar calculation was also made to estimate the THg input per lake volume, in which case the Area was substituted for Volume, representing the total lake volume of each lake (Table S1).

### Estimation of MeHg-sources to Leganubens

As no full MeHg inventory have been made within this study, we estimated and compared various sources of MeHg to Legunabens, i.e. direct wet deposition, surface sediment and stocked fish.

Concentrations in cloud water showed a mean of 0.028 ng L^−1^ MeHg (Sonke, unpublished data, n = 12) and total wet deposition (as recorded at the nearest weather station in the adjacent valley of Bernadouze; Station SAFRAN; x/y coordinates 524000/1753000) during the year of 2014 was 1992 mm. Assuming a constant cloud water concentration and a 100% washout, this would yield a total MeHg direct deposition input of 560 µg MeHg to Legunabens during the year of 2014 (excluding any surface runoff and watershed input). Surface sediment samples (0–2 cm), collected from Legunabens in 2014, showed a THg concentration of 0.149 µg g^−1^ (Hansson, unpublished data, n = 4) and assuming a 3% MeHg content, based on reported data in Bravo *et al*.^77^, this would yield a concentration of 0.004 µg g^−1^. Further, a total of 1320 brown trout were stocked in Legunabens in 2014 (Fig. 1). Assuming an average weight of 3.5 g and a bodily THg concentration of 38 ng g^−1^ w.w., of which near 100% would be in the form of MeHg^68^, this would yield a total MeHg input of 178 µg MeHg. Combined, this would give a total of 738 µg MeHg in Legunabens of which 24% would be MeHg introduced via stocking of brown trout.

### Ethics

The authors declare that collection and sacrifice of all fish included in this study was performed in accordance to local guidelines and regulations, i.e. authorized by Direction départementale des territoires de l’Ariège, with input from Fédération de l’Ariége de pêche et de protection du milieu aquatique and service départemental de l’Office national de l’eau et des milieu aquatiques.

## Electronic supplementary material

Supplementary Information
