# Supplementary material for: Transfer of marine mercury to mountain lakes
Source: Sci Rep. 2017 Oct 5;7:12719. doi: 10.1038/s41598-017-13001-2 (PMC5629254; doi:10.1038/s41598-017-13001-2)
Supplement: Supplementary file 1 — Supplementary Information [file 41598_2017_13001_MOESM1_ESM.pdf]

Supporting information

TRANSFER OF MARINE MERCURY TO MOUNTAIN LAKES

Sophia V. Hansson[1,5*], Jeroen Sonke[2], Didier Galop[3+], Gilles Bareille[4+], Séverine Jean[1], and Gaël Le Roux[1]

[1]EcoLab, Université de Toulouse, CNRS, INPT, UPS, Avenue de l'Agrobiopole, 31326 Castanet Tolosan, France
[2]Observatoire Midi-Pyrénées, Laboratoire Géosciences Environnement Toulouse, CNRS; IRD; Université de Toulouse, 14, Avenue Édouard Belin, 31400 Toulouse, France
[3]Laboratory GEODE UMR 5602, Labex DRIIHM (OHM Haut Vicdessos), CNRS, Université J. Jaurès, 5 Allée A. Machado, 31058 Toulouse, France
[4]CNRS/Univ Pau & Pays Adour, Institut des Sciences Analytiques et de Physico-Chimie pour l'Environnement et les Materiaux – UMR5254, 64000, Pau, France
[5]Current address: Department of Bioscience, Aarhus University, Fredriksborgvej 399, 4000 Roskilde, Denmark

* Contact information;
E-mail: sophia.hansson@bios.au.dk
tel.: +45 8715 86 77

8 pages

3 tables

2 figures

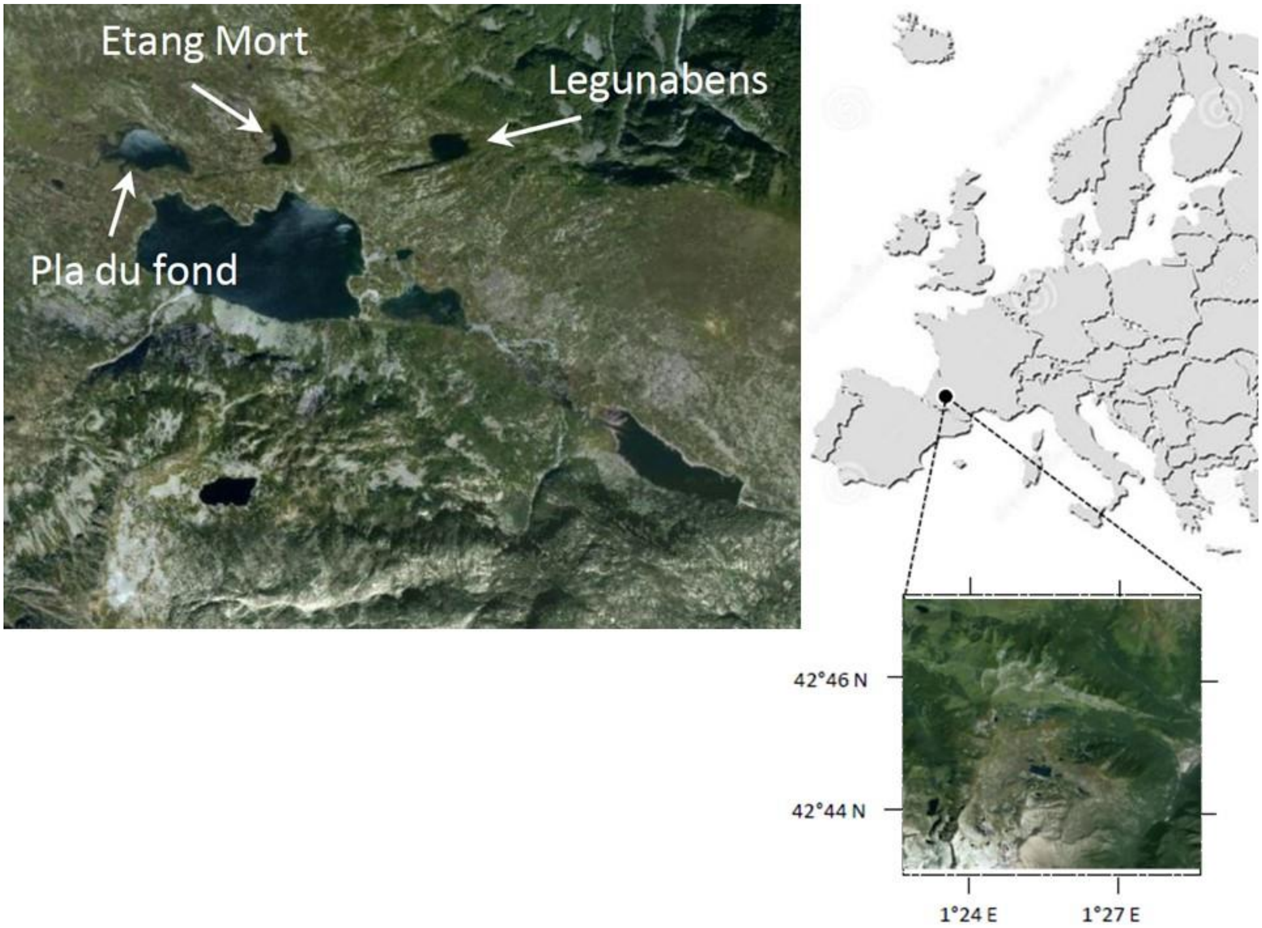

Figure S1. Location of study sites. The map was built under the GIS software QGIS 2.18.11 (https://www.qgis.org)

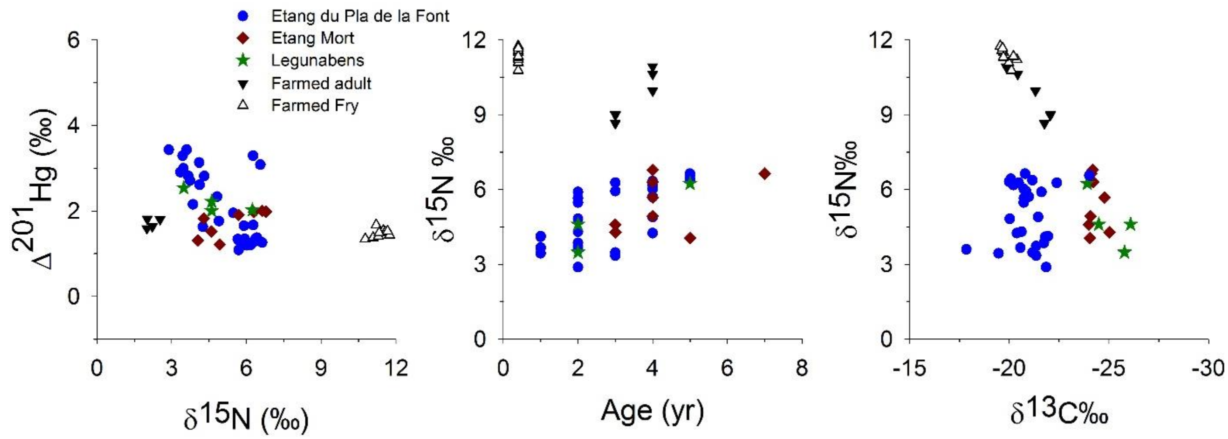

Figure S2 Right) Δ^{199}Hg (‰°) vs δ^{15}N (‰) in muscle tissue. Center) δ^{15}N (‰) in muscle tissue vs age (yr) based on scaliometric analysis. Left) δ^{15}N (‰) vs δ^{13}C (‰). All samples, with the exception of age was measured in muscle tissue of brown trout (*Salmo trutta fario*).

Table S1. Information on study sites.

**Site**	**Etang du Pla de la Font**	**Etang Mort**	**Etang Legunabens**
**GPS X**	42°45’52.79’’N	42°45’52.77’’N	42°45’52.85’’N
**GPS Y**	001°25’09.77’’E	001°25’28.86’’E	001°25’52.25’’E
**Lake surface area**	2.9 ha	0.9 ha	1.0 ha
**Lake volume**	47804 m^3	12453 m^3	23754 m^3
**Elevation a.s.l.**	1653 m	1676 m	1675 m

Table S2. Information on ecological parameters in studied fish.

Site	Fish ID #	Weight (g)	Length (cm)	Age (year)	Sex	Origin
Pla de la Fond	# 1	65,5	17,8	1+	Male	Wild
Pla de la Fond	# 2	28,8	13,8	2+	Female	Wild
Pla de la Fond	# 3	50,4	16,6	2+	Female	Wild
Pla de la Fond	# 4	233,2	28,7	4+	Female	Wild
Pla de la Fond	# 5	66,5	18	1+	Female	Wild
Pla de la Fond	# 6	72,7	18,3	3+	Male	Wild
Pla de la Fond	# 7	151,7	24,7	2+	Female	Wild
Pla de la Fond	# 8	155,6	24,3	4+	Male	Wild
Pla de la Fond	# 9	295,3	31,5	4+	Female	Wild
Pla de la Fond	# 10	241,8	28	5+	Male	Wild
Pla de la Fond	# 11	405	34,1	4+	Female	Wild
Pla de la Fond	# 12	514,3	37,5	5+	Male	Wild
Pla de la Fond	# 13	81,8	19,3	2+	Female	Wild
Pla de la Fond	# 14	160,7	25	2+	Male	Wild
Pla de la Fond	# 15	90,32	22	2+	Female	Wild
Pla de la Fond	# 16	103,5	20,7	2+	Female	Wild
Pla de la Fond	# 17	275	28,8	3+	Male	Wild
Pla de la Fond	# 18	332,6	34,2	5+	Male	Wild
Pla de la Fond	# 19	332,2	31,5	5+	Female	Wild
Pla de la Fond	# 20	159,2	24,5	4+	Female	Wild
Pla de la Fond	# 21	228,5	26,6	4+	Female	Wild
Pla de la Fond	# 22	185,9	26	3+	Male	Wild
Pla de la Fond	# 23	366,9	32,6	4+	Male	Wild
Pla de la Fond	# 24	91,6	20,6	2+	Male	Wild
Pla de la Fond	# 25	328	32,1	4+	Female	Wild
Pla de la Fond	# 26	344,6	32	4+	Female	Wild
Pla de la Fond	# 45	41,4	15,2	2+	Female	x
Pla de la Fond	# 47	64,3	17,4	3+	Female	x
Pla de la Fond	# 54	52,7	17,2	1+	Female	x
Pla de la Fond	# 55	51,6	17,1	1+	Male	x
Etang Mort	# 27	358,7	30,5	4+	Male	Stocked
Etang Mort	# 28	258,7	27,5	4+	Male	Stocked
Etang Mort	# 30	392,8	31,4	4+	Male	Stocked
Etang Mort	# 31	255	27	3+	Female	Stocked
Etang Mort	# 42	253	27,4	5+	Female	x
Etang Mort	# 43	107,8	20,9	4+	Female	x
Etang Mort	# 44	810	39,8	7+	Female	x
Etang Mort	# 53	195,4	23,9	3+	Female	x
Legunabens	# 29	138,2	21	2+	Male	Stocked
Legunabens	# 32	437	33,7	5+	Male	Stocked
Legunabens	# 33	217,8	25,5	2+	Female (?)	Stocked
Legunabens	# 52	100,3	20,8	2+	Male	x
Fish Farm	# 36	1355	46,5	4+	Female	Farmed
Fish Farm	# 37	785	39,3	4+	Female	Farmed
Fish Farm	# 38	1286	43,5	4+	Male	Farmed
Fish Farm	# 39	349	31	3+	Male	Farmed

Fish Farm	# 40	516	33,8	3+	Female	Farmed
Fish Farm	# 41	390	31	3+	Male	Farmed
Fish Farm	Alevin 1	0,67	x	3 months	x	Farmed
Fish Farm	Alevin 2	0,87	x	3 months	x	Farmed
Fish Farm	Alevin 3	1,44	x	3 months	x	Farmed
Fish Farm	Alevin 4	0,74	x	3 months	x	Farmed
Fish Farm	Alevin 5	0,84	x	3 months	x	Farmed
Fish Farm	Alevin 6	0,78	x	3 months	x	Farmed
Fish Farm	Alevin 7	0,52	x	3 months	x	Farmed
Fish Farm	Alevin 8	0,99	x	3 months	x	Farmed
Fish Farm	Alevin 9	1,07	x	3 months	x	Farmed
Fish Farm	Alevin 10	0,84	x	3 months	x	Farmed

Table S3. Information on THg (ng g^{-1} wet weight) and isotopic data of N, C and Hg (‰) as measured in fish muscle tissues.

Identite Poisson	Site	Hg ng g^{-1} w.w.	$\delta^{15}N$	$\delta^{13}C$	N%	C%	$\delta^{199}Hg$	$\delta^{200}Hg$	$\delta^{201}Hg$	$\delta^{202}Hg$	$\delta^{204}Hg$	$\Delta^{199}Hg$	$\Delta^{200}Hg$	$\Delta^{201}Hg$	$\Delta^{204}Hg$
**# 1**	Pla de la Fond	165,63	4,12	-21,94	13,73	45,18	2,61	0,09	2,10	0,00	-0,20	2,61	0,09	2,10	-0,20
**# 2**	Pla de la Fond	82,79	2,88	-21,85	14,09	45,73	3,47	0,17	2,99	0,17	0,37	3,43	0,09	2,87	0,12
**# 3**	Pla de la Fond	160,22	3,73	-21,35	14,86	47,09	2,71	0,01	2,28	0,00	-0,05	2,71	0,01	2,28	-0,06
**# 4**	Pla de la Fond	593,20	6,27	-22,38	13,99	46,75	3,26	0,05	2,48	-0,10	-0,34	3,29	0,10	2,56	-0,19
**# 5**	Pla de la Fond	176,03	4,10	-21,81	14,20	44,83	3,14	0,11	2,55	0,03	-0,06	3,13	0,10	2,53	-0,10
**# 6**	Pla de la Fond	103,57	3,35	-21,35	14,12	46,52	2,93	0,22	2,49	0,11	0,12	2,90	0,17	2,40	-0,05
**# 7**	Pla de la Fond	205,09	5,65	-20,73	13,78	45,98	1,28	0,05	0,92	-0,21	-0,58	1,34	0,16	1,08	-0,26
**# 8**	Pla de la Fond	222,95	4,89	-21,45	13,59	47,49	1,68	-0,05	1,24	-0,32	-0,71	1,76	0,11	1,48	-0,23
**# 9**	Pla de la Fond	434,26	6,33	-20,00	13,31	46,10	1,21	-0,03	0,85	-0,30	-0,75	1,29	0,12	1,07	-0,30
**# 10**	Pla de la Fond	475,71	6,43	-20,08	14,23	46,04	1,31	0,06	0,90	-0,23	-0,63	1,37	0,18	1,08	-0,28
**# 11**	Pla de la Fond	532,73	6,17	-20,21	14,09	46,95	1,10	-0,09	0,66	-0,42	-0,63	1,21	0,12	0,97	-0,01
**# 12**	Pla de la Fond	442,87	6,63	-20,79	13,83	47,14	1,15	-0,20	0,74	-0,43	-0,63	1,26	0,01	1,06	0,00
**# 13**	Pla de la Fond	148,45	4,30	-20,61	14,27	46,37	2,83	0,15	2,43	0,08	-0,08	2,81	0,11	2,37	-0,20
**# 14**	Pla de la Fond	319,50	5,90	-21,61	14,36	46,13	1,58	-0,11	1,11	-0,29	-0,54	1,65	0,03	1,33	-0,11
**# 15**	Pla de la Fond	427,13	5,48	-20,72	14,00	47,44	1,90	-0,01	1,36	-0,19	-0,47	1,95	0,08	1,51	-0,19
**# 16**	Pla de la Fond	268,90	4,82	-20,02	14,36	45,98	2,31	-0,01	1,84	-0,11	-0,26	2,33	0,04	1,92	-0,10
**# 17**	Pla de la Fond	302,40	6,28	-20,47	13,49	45,48	1,59	-0,05	1,12	-0,32	-0,82	1,67	0,11	1,37	-0,34
**# 18**	Pla de la Fond	527,52	6,56	-24,02	14,76	45,60	3,07	0,06	2,32	-0,04	-0,27	3,08	0,08	2,35	-0,22
**# 19**	Pla de la Fond	305,25	6,37	-21,16	12,49	51,54	1,27	-0,06	0,81	-0,33	-0,79	1,35	0,10	1,06	-0,30
**# 20**	Pla de la Fond	100,58	4,25	-20,38	14,65	45,92	1,58	0,01	1,21	-0,21	-0,34	1,63	0,11	1,36	-0,04
**# 21**	Pla de la Fond	232,83	5,71	-20,98	14,09	47,11	1,22	-0,04	0,82	-0,32	-0,63	1,30	0,12	1,06	-0,15
**# 22**	Pla de la Fond	275,79	5,93	-20,84	13,91	46,56	1,25	-0,05	0,77	-0,37	-0,68	1,35	0,13	1,04	-0,13
**# 23**	Pla de la Fond	365,62	6,31	-19,99	14,13	46,56	1,22	0,01	0,81	-0,23	-0,64	1,28	0,12	0,98	-0,29
**# 24**	Pla de la Fond	112,25	3,85	-21,74	14,66	46,95	2,15	0,07	1,83	-0,02	-0,06	2,15	0,08	1,85	-0,03
**# 25**	Pla de la Fond	361,42	6,02	-20,72	12,66	45,27	1,12	-0,04	0,64	-0,31	-0,55	1,20	0,11	0,88	-0,09

**# 26**	Pla de la Fond	324,08	5,69	-20,77	13,58	45,59	0,99	-0,12	0,68	-0,36	-0,64	1,08	0,06	0,95	-0,11
**# 45**	Pla de la Fond	60,22	3,60	-17,85	15,30	49,94	3,65	0,54	3,40	0,86	1,05	3,43	0,11	2,75	-0,23
**# 47**	Pla de la Fond	115,13	3,47	-21,17	15,05	50,95	3,12	0,37	2,77	0,51	0,62	2,99	0,11	2,38	-0,14
**# 54**	Pla de la Fond	68,99	3,67	-20,55	15,75	50,78	3,00	0,45	2,76	0,71	0,66	2,82	0,09	2,23	-0,40
**# 55**	Pla de la Fond	78,20	3,44	-19,46	15,51	50,32	3,46	0,44	3,14	0,68	0,64	3,29	0,10	2,63	-0,37
**# 27**	Etang Mort	679	6,30	-24,21	13,92	47,21	1,90	-0,10	1,25	-0,30	-0,41	1,97	0,05	1,47	0,03
**# 28**	Etang Mort	385	5,68	-24,78	13,95	46,25	1,83	-0,01	1,22	-0,28	-0,66	1,90	0,13	1,43	-0,24
**# 30**	Etang Mort	780	6,79	-24,19	14,58	48,31	1,92	-0,05	1,33	-0,24	-0,38	1,98	0,08	1,51	-0,02
**# 31**	Etang Mort	183	4,28	-25,03	14,39	46,81	1,70	-0,08	0,99	-0,46	-0,86	1,82	0,15	1,33	-0,18
**# 42**	Etang Mort	75	4,05	-24,04	14,81	53,69	1,36	0,22	1,21	0,19	0,14	1,31	0,13	1,07	-0,14
**# 43**	Etang Mort	87	4,93	-24,08	14,91	50,90	1,29	0,31	1,16	0,29	0,08	1,21	0,17	0,95	-0,35
**# 44**	Etang Mort	514	6,64	-24,13	14,55	53,91	1,97	0,03	1,43	-0,14	-0,37	2,00	0,10	1,53	-0,17
**# 53**	Etang Mort	190	4,59	-23,99	15,21	50,65	1,53	0,13	1,15	0,05	-0,21	1,51	0,10	1,11	-0,29
**# 29**	Legunabens	114,24	4,61	-26,09	13,74	47,72	2,24	0,13	1,69	0,06	-0,18	2,22	0,10	1,64	-0,28
**# 32**	Legunabens	229,28	6,24	-23,91	14,21	46,83	1,96	0,05	1,46	-0,16	-0,33	2,00	0,13	1,58	-0,09
**# 33**	Legunabens	126,01	4,60	-24,49	14,57	46,41	1,99	0,01	1,35	-0,13	-0,43	2,02	0,08	1,45	-0,23
**# 52**	Legunabens	63,71	3,48	-25,79	14,34	47,59	2,64	0,36	2,21	0,41	0,37	2,53	0,15	1,91	-0,24
**# 36**	Hatchery	216,06	9,95	-21,31	14,08	54,69	1,79	0,25	1,75	0,61	0,86	1,63	-0,06	1,29	-0,05
**# 37**	Hatchery	155,63	10,93	-19,83	14,87	49,77	1,73	0,31	1,71	0,56	0,84	1,59	0,03	1,29	0,00
**# 38**	Hatchery	154,75	10,63	-20,42	14,11	51,93	1,86	0,19	1,54	0,22	0,30	1,81	0,08	1,38	-0,03
**# 39**	Hatchery	157,88	8,65	-21,76	14,65	51,77	1,86	0,20	1,60	0,24	0,36	1,80	0,08	1,42	0,01
**# 40**	Hatchery	193,45	9,02	-22,07	14,09	54,10	2,09	0,48	1,89	0,62	0,98	1,94	0,17	1,42	0,06
**# 41**	Hatchery	215,07	8,99	-22,06	13,84	53,32	2,04	0,29	1,97	0,57	1,06	1,90	0,01	1,54	0,22
**Alevin 1**	Hatchery	28,95	10,77	-20,10	11,71	47,85	1,46	0,22	1,37	0,48	0,92	1,34	-0,02	1,01	0,21
**Alevin 2**	Hatchery	18,79	11,08	-19,97	12,16	49,93	1,56	0,35	1,69	0,74	0,88	1,37	-0,02	1,13	-0,22
**Alevin 3**	Hatchery	19,27	11,21	-20,37	11,78	51,47	1,84	0,47	1,75	0,69	0,73	1,67	0,12	1,23	-0,29
**Alevin 4**	Hatchery	18,08	11,50	-19,71	13,14	49,89	1,64	0,18	1,64	0,41	0,86	1,53	-0,03	1,33	0,25

**Alevin 5**	Hatchery	28,37	11,57	-19,63	12,30	47,03	1,58	0,12	1,52	0,32	0,98	1,50	-0,04	1,28	0,50
**Alevin 6**	Hatchery	19,94	11,75	-19,55	13,17	48,65	1,57	0,27	1,75	0,53	0,94	1,43	0,00	1,35	0,15
**Alevin 7**	Hatchery	5,14	11,48	-19,60	12,78	47,83	x	x	x	x	x	x	x	x	x
**Alevin 8**	Hatchery	19,43	11,30	-19,70	12,71	49,11	1,52	0,20	1,64	0,46	0,60	1,41	-0,03	1,30	-0,09
**Alevin 9**	Hatchery	15,94	11,33	-20,21	12,15	50,82	1,59	0,11	1,69	0,41	0,75	1,49	-0,10	1,38	0,14
**Alevin 10**	Hatchery	20,60	11,67	-19,66	12,91	49,49	1,63	0,28	1,61	0,43	0,52	1,53	0,06	1,28	-0,12
